# Injudicious antibiotic use leading to fulminating *Clostridium difficile* infection: a case report

**DOI:** 10.4076/1757-1626-2-7978

**Published:** 2009-09-15

**Authors:** Sreedhar Kolli, Akhila Mallipedhi, Rhys Thomas, Male Kishore Reddy

**Affiliations:** 1 Department of Rehabilitation Medicine, Rookwood Hospital, Fairwater Road, Cardiff, CF5 2YN, UK; 2 Department of Endocrinology, Singleton Hospital, Sketty lane, Sketty, Swansea, SA2 8QA, UK; 3 Department of Neurology, University Hospital of Wales, Heath Park, Cardiff, CF14 4XW, UK; 4 Department of Accident and Emergency Medicine, University Hospital of Wales, Heath Park, Cardiff, CF14 4XW, UK

## Abstract

The case illustrates the myriad of fulminating complications due to *Clostridium difficile* infection in a previously healthy individual without any risk factors. Community acquired *Clostridium difficile* infection can occur even many weeks after the course of broad spectrum antibiotics. There is no definitive pattern or guidelines to predict who would develop the fulminating complications.

## Case presentation

A previously fit 67-year-old, British, Caucasian retired engineer was assisting in heavy manual labour when he sustained a crush injury to his big toe. He had seen many of these managed conservatively by the medical profession before, so it was two weeks before he sought medical assistance. He visited his general practioner (GP) when the nail was lifting off, concerned that it may be infected. This gentleman was admitted into hospital where the nail was surgically removed and he was given a week's course of flucloxacillin. He attended emergency department every other day to have the dressings changed and in time it slowly healed.

Four to six weeks later he began to suffer from loose stools. Again he managed this at home, initially with Imodium and then with codeine from his GP. After about a week, these agents failed to control his motions and he noted that the odour was now more offensive. Stool cultures were negative for *Shigella*, *Salmonella*, *E. coli* and *Campylobacter*. He saw his GP again, and was now showing signs of dehydration, so he was admitted to hospital, isolated and put onto intravenous fluids and metronidazole. Blood tests demonstrated acute renal failure with systemic sepsis.

Four days later, stool cultures confirmed the presence of *Clostridium difficile*. He was now opening his bowels every half an hour day and night. Fluid resuscitation continued and his abdominal plain films showed grossly dilated loops of bowel. After a week, vancomycin and dietary supplements were added and the team began to liase with their surgical colleagues as his symptoms had not abated. After twenty days he was so hypoalbuminaemic that his peripheral oedema hampered peripheral access; therefore nasogastric feeding was commenced. He continued to deteriorate and the medical and surgical teams were concerned about that he had a persistent megacolon and gross colonic oedema seen on sigmoidoscopy. After considering the options carefully, a defunctioning ileostomy was created following a total colectomy, four weeks after admission.

He came round from the operation with his left foot in extreme pain. It was noted to be cool, dusky and have inpalpable distal pulses and found to have an arterial clot. This failed to improve and required a femoral-peroneal bypass. His post-op recovery was further complicated by the need for intensive dietary support as his pressure areas began to ulcerate. His wounds were infected with MRSA, but did not ulcerate. A month later, he noticed that his left foot was cooler again. To his dismay the surgeons confirmed that the graft had occluded and so he went on to have a left below-knee amputation. This wound was again complicated by MRSA infection. After time at a community hospital he was discharged home, sixteen weeks after admission with complete recovery and followed up in the prosthetic clinic.

***Clostridium difficile* - The enemy within.** Since 1977 *Clostridium difficile* has been recognised as the cause of pseudomembranous colitis. The bacterium is an anaerobic, toxigenic, gram-positive rod. It produced spores which can survive for months on surfaces, with many people remaining asymptomatic carriers. The role of antibiotics in disrupting normal bowel flora and therefore predisposing to infection has been well described before.

Swift identification and then removal of precipitating factors are the initial parts of treatment. Oral metronidazole or vancomycin alongside fluid and electrolyte replacement is often enough to improve symptoms. Patients who fail to respond need dietary supplementation, consideration of live yoghurts and withholding Proton pump inhibitors and early surgical review. Despite all this intervention, recurrence is common. This emphasises the current focus on initial prevention.

## Discussion

Community acquired *Clostridium difficile* diarrhoea (CACDD) is defined as diarrhoea developed outside hospital settings. Although there is no universally agreed definition for CACDD, various studies have taken various criteria ranging from less than 72 hours to 6 months after exposure to antibiotics [1,2]. However, CACDD can occur in a individuals with no recent exposure to antibiotics [3,5]. The community and hospital acquired infections are both interlinked. Recent studies in Canada, USA and Europe has showed CACDD is rising [6].Current trends show the risk factors are also changing [7]. Clostridium difficile infection in the UK causes an average increased stay of 21 days and has an estimated cost of £4,000 per case [8]. Fulminant CDAD is also increasing. It is a state of clostridium difficile colitis not responsive to medical therapy. It is complicated by leukocytosis (peripheral white blood cell count >16 000/mm^3^), hypoalbuminemia, renal failure and emergency colectomy and even death.

This man initially had a mechanical injury to his toe and now has a stoma and an amputation. He was originally fourteen stone of muscle, able to lift a breezeblock with one hand. At one stage of his rehabilitation, he struggled to lift the bottle of Lucozade, which he was using as a dumb-bell. This man was desperately unlucky to have such an aggressive strain of *C. Diff* colitis, as he was not immunocompromised and had no comorbidities. He developed most of the recognised complications; surgery prevented colonic perforation. The incidence of this is increased by the use of anti-motility preparations, which was his initial strategy in the community.

Antibiotic resistance and prescription cost reduction were the drivers in teaching temperance in antibiotic use. The risks of allergy and pseudomembranous colitis are also not new phenomena. However *Clostridium* now is not uniquely a disease of the elderly, no longer victimises only the frail and the chronicity of antibiotic consumption and symptoms can surprise the diagnostic.

His case is a reminder of our part in the butterfly effect: Even the smallest of actions may have greater implications, that were initially unforeseen.

## Consent

Written informed consent was obtained from the patient for publication of this case report. A copy of this written consent is available for review by the Editor-in-chief of this journal.

## Competing interests

The authors declare that they have no competing interests.

## Authors' contributions

SK gathered data, analyzed data and played a key role in writing the case report. RT and AM edited the English and provided references and proof read the article. MKR actively participated in literature review and writing the case report.
